# Selected Brain Metabolites and Mitochondrial DNA Copy Number as Potential Markers of Ongoing Neurodegeneration in Patients with Wolfram Syndrome

**DOI:** 10.3390/metabo16040281

**Published:** 2026-04-20

**Authors:** Ewa Zmysłowska-Polakowska, Tomasz Płoszaj, Sebastian Skoczylas, Julia Grzybowska-Adamowicz, Dobromiła Barańska, Katarzyna Matera, Aleksandra Palatyńska-Ulatowska, Wojciech Młynarski, Agnieszka Zmysłowska, Michal Ciborowski

**Affiliations:** 1 Department of Endodontics, Medical University of Lodz, Pomorska Str. 251, 92-213 Lodz, Poland; aleksandra.palatynska-ulatowska@umed.lodz.pl; 2 Department of Clinical Genetics, Medical University of Lodz, Pomorska Str. 251, 92-213 Lodz, Poland; tomasz.ploszaj@umed.lodz.pl (T.P.); sebastian.skoczylas@umed.lodz.pl (S.S.); julia.grzybowska-adamowicz@umed.lodz.pl (J.G.-A.); agnieszka.zmyslowska@umed.lodz.pl (A.Z.); 3 Department of Diagnostic Imaging, Polish Mother’s Memorial Hospital-Research Institute, Rzogowska Str. 281/289, 93-338 Lodz, Polandkatarzyna.matera@iczmp.edu.pl (K.M.); 4 Department of Pediatrics, Oncology and Hematology, Medical University of Lodz, Pomorska Str. 251, 92-213 Lodz, Poland; wojciech.mlynarski@umed.lodz.pl; 5 Metabolomics and Proteomics Laboratory, Clinical Research Centre, Medical University of Bialystok, Waszyngtona Str. 17, 15-369 Bialystok, Poland; michal.ciborowski@umb.edu.pl; 6 Department of Medical Biochemistry, Medical University of Bialystok, Mickiewicza Str. 2C, 15-089 Bialystok, Poland

**Keywords:** Wolfram syndrome, MRS, neurodegeneration, mtDNA copy number, markers

## Abstract

**Background**: Wolfram syndrome (WFS) is a rare neurodegenerative disease that is genetically determined and inherited in an autosomal recessive manner. Although the first clinical symptom appearing in early childhood is diabetes mellitus, subsequent symptoms are associated with optic nerve atrophy, followed by central nervous system atrophy. **Methods**: The aim of the study was to analyse magnetic resonance images (MRI) of the brain in combination with single-voxel magnetic resonance spectroscopy (MRS) and to assess the copy number of mitochondrial DNA (mtDNA-CN) in 10 patients with WFS compared with a control group of 17 healthy individuals. **Results**: A significant decrease in the amount of selected metabolites was observed in WFS patients compared to controls in all assessed brain regions (pons, cerebellum, white matter, thalamus, and hippocampus). For three metabolites, Glutamate (Glu), Glutamate + Glutamine (Glx) and total N-acetylaspartate (TNAA), significant differences in concentrations were found between the study groups in almost all matrices evaluating specific areas of the brain (*p* < 0.011), with the exception of a trend toward reduced TNAA in the hippocampus (*p* = 0.065). In addition, patients with WFS had a significant decrease in the mitochondrial-to-nuclear DNA ratio compared to controls (*p* < 0.0003). Some metabolites, such as N-acetylaspartate and total N-acetylaspartate, showed strong correlations with specific regions of the visual pathway on MRI scans in patients with WFS. **Conclusions**: Selected brain metabolites and mtDNA-CN may become potential markers of WFS, and the results of this study may be used to define indicators for future therapeutic strategies.

## 1. Introduction

Wolfram syndrome (WFS) is an ultra-rare neurodegenerative disorder with autosomal recessive inheritance, with an estimated prevalence of 1 in 770,000 [1,2]. Two causative mutations in the *WFS1* gene lead to the loss of wolframin protein function, resulting first in insulin-dependent diabetes in children around 5–7 years of age, followed by a wide spectrum of neurodegenerative disorders, ranging from optic nerve atrophy and cerebellar ataxia to premature death in patients [3,4,5]. These symptoms are also accompanied by other disorders, such as urological, endocrine and psychiatric problems [5,6,7]. In addition, animal models have shown that mice with Wfs1 deficiency exhibit altered serotonergic system function [8]. Efforts are ongoing to identify markers of the clinical course of this syndrome and effective causal treatments for WFS patients [9,10,11,12]. So far, parameters assessed during the optical coherence tomography (OCT) and brain magnetic resonance imaging (MRI) studies have been recognized as important clinical markers of disease progression [13,14,15,16]. New studies using qualitative MRI analysis in WFS patients have expanded the spectrum of white matter changes to include progressive inflammatory demyelination [17]. Longitudinal neuroimaging studies in patients with WFS have identified brain regions that show reductions across various stages of neurodegeneration, including the brainstem, cerebellum, thalamus, and white matter [15,16,17]. Moreover, the LC-MS (Liquid chromatography–Mass spectrometry) method was used to obtain a serum metabolic fingerprint of WFS patients, indicating that sphinganine derivatives may be a marker of ongoing neurodegeneration in these patients [18]. Furthermore, through metabolomic analysis of gingival crevicular fluid (GCF) collected from the oral cavity, metabolites distinguishing WFS patients from those with type 1 diabetes and healthy controls were identified [19]. Therefore, there was still a need for research to identify brain metabolites associated with neurodegeneration in affected brain regions of patients with WFS.

On the other hand, single-voxel magnetic resonance spectroscopy (MRS) is useful both for identifying markers of various neurodegenerative diseases and for monitoring the effectiveness of their treatment [20,21,22]. This relationship also applies to mitochondrial DNA copy number (mtDNA-CN), which is increasingly recognised as a potential marker of various neurodegenerative disorders [23,24]. In the case of WFS, it is particularly interesting that we are dealing with both insulin-dependent diabetes, where the copy number of mtDNA has already been described as an indicator of this disorder, as well as the progression of neurodegeneration [25].

The aim of this study was to compare magnetic resonance images of the brain with spectroscopic assessment (MRS) in patients with Wolfram syndrome and a control group. In addition, in the study group, MRS results were correlated with clinical parameters and recognized neuroimaging parameters, as well as mtDNA-CN.

## 2. Materials and Methods

The study group comprised 10 patients with Wolfram syndrome who participated in the clinical trial “TreatWolfram” (NCT03717909). The study was approved by the relevant bioethics committee (RNN/379/19/KE and RNN/191/19/KE), and all participants and/or their parents signed an informed consent form. The WFS diagnosis in all participants was confirmed by Sanger sequencing to identify causative variants in the *WFS1* gene, as previously described [26]. All WFS patients had insulin-dependent diabetes mellitus diagnosed based on WHO criteria, coexisting with optic nerve atrophy supported by the OCT method. Clinical parameters assessed in patients with WFS included: indicators of glucose metabolism (glucose level and glycated hemoglobin—HbA1c), patients’ age at diagnosis, body weight, and BMI (body mass index).

Data obtained from patients with WFS were compared with data from a control group of healthy individuals (n = 17) matched for gender (*p* = 0.41) and age (*p* = 0.36); the mean age of patients was 24.6 ± 4.12 years, whilst in the control group it was 22.6 ± 6.82 years.

Brain MRI with spectroscopic evaluation was performed on individuals from the study and control groups, and a buccal mucosa swab was collected to assess mtDNA-CN.

### 2.1. Magnetic Resonance Spectroscopy (MRS) Analysis

All scans were conducted on a 3.0 T Achieva dSTREAM scanner (Philips, Cambridge, MA, USA). Initially, a three-dimensional T1-weighted turbo field echo (3D TFE) sequence was acquired to enable precise planning of the MRS voxels. Proton MRS measurements were then performed using a single-voxel (SV) acquisition technique. The following SV PRESS sequence parameters were implemented in the examinations of our patients: TE = 35 ms, TR = 2000 ms, NSA = 128, flip angle: 90°, spectral resolution: 1.95 Hz/point, spectral bandwidth: 2000 Hz, samples: 1024. The voxel geometry was adapted to the anatomical region and comprised cubic, cuboidal or trapezoidal shapes. The minimum voxel edge length was 10 mm, and the maximum was 20 mm. Voxel volumes ranged from 1 to 8 mL.

For each participant, one voxel was positioned in each of the following brain regions: pons, cerebellar hemisphere white matter (cerebellum), cerebral hemisphere white matter (white matter), hippocampus and thalamus.

For anatomically symmetrical structures, voxel placement was performed primarily on the left side of the brain and cerebellum. If left-sided placement was prevented by technical or anatomical constraints, the voxel was positioned on the contralateral side. Each MRS sequence was performed twice per patient, with and without water saturation. Following data acquisition, the raw MRS spectra were exported to Tarquin (version 4.3.10), an open-source software package for automated spectral analysis. Quantification of neural tissue metabolites was carried out fully automatically using the default Tarquin processing pipeline, as described previously [27].

To assess visual pathway atrophy, two experienced radiologists independently measured the transverse diameter of the intracranial and intraorbital portions of the optic nerves, the optic chiasm, and the optic tract on volumetric T1-weighted images in axial, coronal, and sagittal planes, as described earlier [28].

### 2.2. Analysis of the mtDNA Copy Number

From patients and the control group, cheek mucosal swab samples were collected non-invasively during routine dental examinations performed by two experienced dentists. Samples for molecular analysis were collected in sterile vials with screw caps and then stored at −20 °C.

Total DNA (from both the nuclear and mitochondrial genomes) was extracted from swabs using QIAamp DNA kits (QIAGEN, Hilden, Germany) according to the manufacturer’s protocol. The copy number of mtDNA was quantified using quantitative PCR (qPCR) assay performed on a LightCycler 480 instrument (Roche Diagnostics, Rotkreuz, Switzerland) with TaqMan probes (Thermo Fisher Scientific, Waltham, MA, USA). The *MT-TL1* gene was amplified as the mtDNA target, as previously described by Rooney et al. [29]. The beta-globin gene was used as an internal reference and was also detected using a TaqMan probe. Both targets were amplified in the same reaction using different dyes, in triplicate, under the manufacturer’s standard cycling conditions for the TaqMan™ Genotyping Master Mix (Thermo Fisher Scientific, Waltham, MA, USA). The primers and conditions are shown in Supplementary Table S1. The relative abundance of mitochondrial DNA was calculated using the ΔCt method, where ΔCt = Ct_mtDNA − Ct_nDNA, and the relative quantities were derived as R = 2^−ΔCt^.

### 2.3. Statistical Analysis

Prior to statistical analysis, the data matrix of brain metabolites was filtered to include only metabolites measured in at least 80% of patients. All metabolites were normalized to TCr (total creatine) prior to analysis. Where values were missing, the k-Nearest Neighbors (KNN) method was used to impute the data. Clustering with a heat map and a violin plot was performed for each brain region, indicating statistically significant values (Mann–Whitney U test) adjusted by the false discovery rate (FDR) correction. Cohen’s d method was used to estimate the effect size in the post hoc power analysis. Statistical analysis for mtDNA-CN assessment was performed using the Shapiro–Wilk test to assess the normality of each group and Levene’s test to assess homogeneity of variance. Due to unequal variances, group comparisons were performed using Welch’s *t*-test. Next, for the group of patients with WFS, Spearman’s correlation analysis was performed between the metabolites measured in MRS, clinical and neuroimaging parameters, and mtDNA-CN. Statistical significance was defined as *p* < 0.05.

## 3. Results

**Figure 1:**
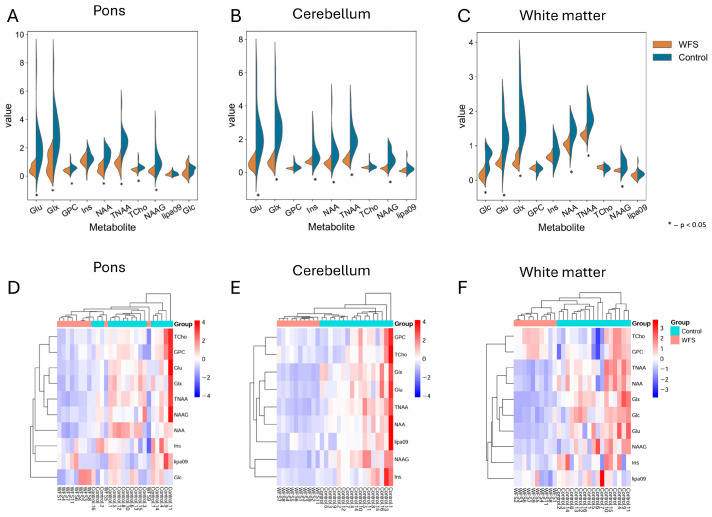
The figure shows metabolite concentrations on violin plots (**A**–**C**) and heatmaps for the same data (**C**–**E**). Graphs (**A**,**D**) present values for the pons; graphs (**B**,**E**) present values for the cerebellum and graphs (**C**,**F**) present values for the white matter of the brain in both analyzed groups. The graphs only include metabolites for which at least 80% of the MRS data were obtained. An asterisk indicates differences in metabolites where *p* < 0.05; WFS—Wolfram syndrome.

Figure 1A shows that the concentrations of eight metabolites—Glu (Glutamate), Glx (Glutamate + Glutamine), GPC (glycerophosphocholines), Ins (myo-inositol), NAA (N-acetylaspartate), TNAA (total N-acetylaspartate), Tcho (total Choline) and NAAG (N-acetylaspartylglutamate)—in the pons were lower in the WFS group than in the control group (all *p* values < 0.012). No significant differences were observed for Glc (Glucose) and Lipa09 (mobile lipids) in this brain region (all *p* values *p* > 0.143).

Six out of nine metabolites in the cerebellum (Figure 1B) showed significant differences (Glu, Glx, Ins, NAA and TNAA, as well as NAAG), with concentrations that were higher in the control group than in the WFS group (all *p* values < 0.005). No such differences were shown for three metabolites: GPC, Tcho and Lipa09 (all *p* values *p* > 0.128).

However, six out of ten metabolites in white matter (Figure 1C) showed significant differences (Glu, Glx, NAA, TNAA, NAAG and Glc), with concentrations also higher in the control group than in the WFS group (all *p* values < 0.012). No such differences were shown for four metabolites: GPC, Ins, Tcho and Lipa09 (all *p* values *p* > 0.116).

It should be emphasized that NAAG showed a significant reduction in WFS patients compared to the control group in all three brain regions (pons, cerebellum and white matter), as shown in Figure 1 (all *p* < 0.012) (Figure 1A–C, and Supplementary Table S2). One of the key metabolites in diabetes, Glc, showed significant differences (*p* = 0.002) in the white matter (Figure 1C), whilst no such difference (*p* = 0.459) was observed in the pons (Figure 1A). A post hoc statistical power analysis yielded values > 0.5 for the vast majority of metabolites. In addition, looking at the heatmaps clustering, we observe that the control group is almost perfectly separated from the study group, based on metabolites measured in the cerebellum and white matter (Figure 1E,F), which cannot be stated in relation to the pons region (Figure 1D).

**Figure 2:**
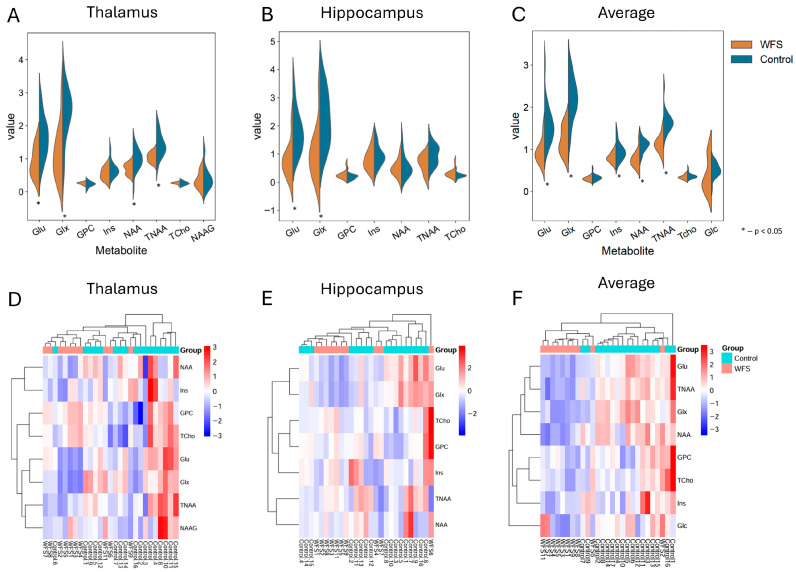
The figure shows metabolite concentrations on violin plots (**A**–**C**) and heatmaps for the same data (**C**–**E**). Graphs (**A**,**D**) present values for the thalamus; graphs (**B**,**E**) present values for the hippocampus and graphs (**C**,**F**) present average data obtained for all brain regions in both analyzed groups. The graphs only include metabolites for which at least 80% of the MRS data were obtained. An asterisk indicates differences in metabolites where *p* < 0.05; WFS—Wolfram syndrome.

Figure 2 shows the values for two regions, the hippocampus and the thalamus, as well as the mean values for all brain regions. Four of eight metabolites in the thalamus (Figure 2A) showed significant differences (Glu, Glx, NAA, and TNAA), with higher concentrations in the control group than in the WFS group (all *p* values < 0.015). No such differences were shown for four metabolites: Ins, GPC, NAAG and Tcho (all *p* values > 0.206).

In the hippocampus (Figure 2B), two of the seven metabolites (Glu/Glx) exhibited significantly higher concentrations in the control group than in the WFS group (all *p* values < 0.011). However, no such differences were observed for GPC, Ins, NAA, TCho and 8TNAA (all *p* values *p* > 0.065).

Significant differences in Glu and Glx metabolites were observed in both brain regions (thalamus and hippocampus), as shown in Figure 2A,B (all *p* values < 0.011). The mean values for all brain regions (Figure 2C) indicated that five of the eight metabolites (Glu, Glx, Ins, NAA, and TNAA) were present in lower concentrations in the WFS group (all *p* values < 0.009). No differences were observed in the mean concentrations for GPC, Tcho, and Glc (all *p* values > 0.122).

The heatmaps in Figure 2D,E suggest that these brain regions do not differ perfectly between the study groups. Figure 2F shows a comprehensive heat map of mean values that separates the WFS group from the control group, but this separation is not perfect.

In summary, significant differences in the concentrations of three metabolites (Glu/Glx and TNAA) were found in almost all matrices when evaluating specific brain areas between the study group and the control group (*p* < 0.011), except for TNAA in the hippocampus, where the *p*-value was slightly above the 0.05 threshold (*p* = 0.065). In contrast, no significant changes were observed for the metabolite Lipa09 (all *p*-values > 0.128) (see Figure 1 and Figure 2 and Supplementary Table S2).

**Figure 3:**
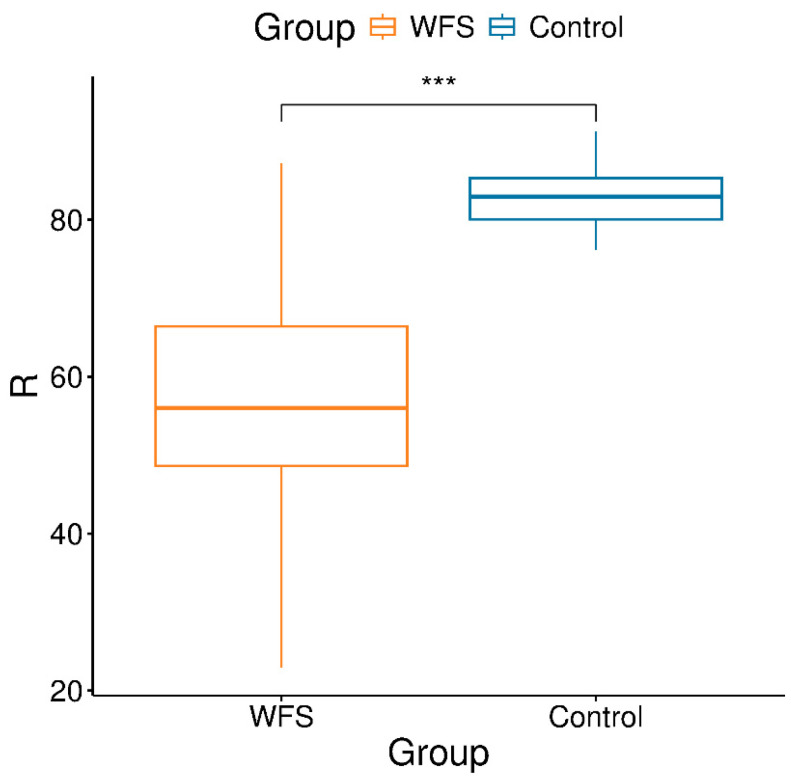
Distribution of R values (mitochondrial-to-nuclear DNA ratio) in WFS and control groups (*** *p* < 0.0003).

Next, an analysis comparing mitochondrial DNA copy numbers between groups showed a significant reduction in the mitochondrial-to-nuclear DNA ratio in patients with WFS (Welch’s t = −5.04, df = 12.67, *p* = 2.45 × 10^−4^). The mean R value in the study group was lower than in the control group, indicating a greater depletion of mitochondrial genome copies relative to the nuclear genome (Figure 3).

**Figure 4:**
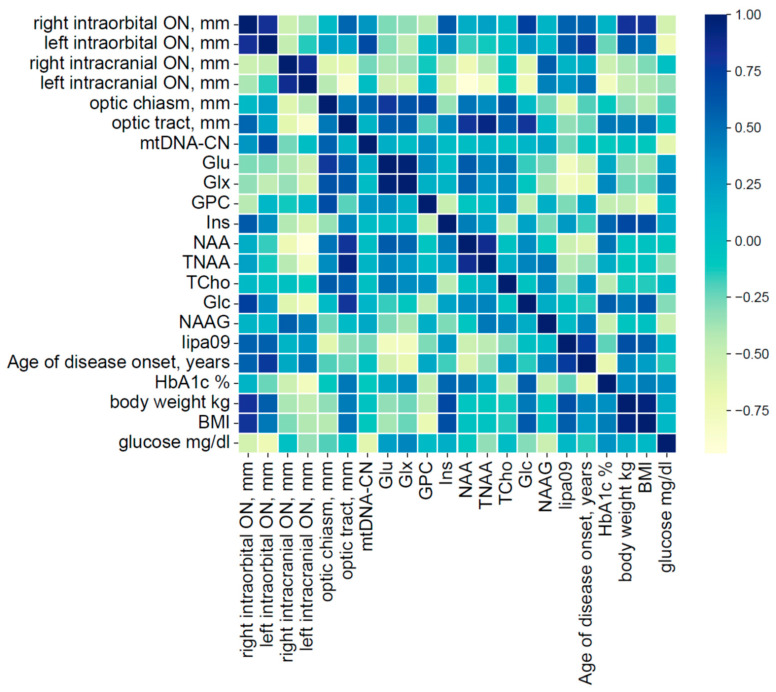
Correlation coefficient plot for mean metabolite concentrations, mtDNA copy number, MRI-optic pathway measurements and clinical data in WFS patients’ group.

Furthermore, correlations between brain MRS metabolites and mtDNA-CN, as well as both MRI markers of neurodegeneration (Figure 4 and Table 1) and clinical parameters (Figure 4 and Table 2), were assessed in patients with WFS.

Statistically significant strong correlations were found between NAA level and the left intracranial part of the optic nerve and between TNAA and optic tract thickness (respectively, *p* = 0.005 and *p* = 0.011; Table 1). Significant correlations were also found between the age of disease diagnosis (diabetes mellitus as the first symptom of Wolfram syndrome) and Glx and lipa09 (*p* = 0.042 and *p* = 0.015, respectively) (Table 2). Furthermore, the body weight of patients with WFS correlated strongly with Ins (*p* = 0.036) and BMI with GPC levels (*p* = 0.032) (Table 2).

Unfortunately, mtDNA-CN did not correlate significantly with MRI or clinical parameters of the visual pathway, but a tendency toward correlation was observed with the left intraorbital portion of the optic nerve (*p* = 0.126).

## 4. Discussion

This study was the first to evaluate MRS in patients with WFS. The analysis revealed lower levels of several metabolites in neural tissue in patients with WFS compared to the control group. Importantly, most of the changes in metabolite concentrations were found in the cerebellum, pons, and white matter, which correspond with the neurodegenerative process specific to WFS. A common and early symptom of neurodegeneration observed in patients with WFS is ataxia [6,30].

Interestingly, Glx and Glu levels were decreased in all five regions studied. Glu is a well-known neurotransmitter, and the entire Glx component has been called a “marker of neuro-excitotoxicity”, while Glu is mainly involved in Glx synthesis [31,32]. A decrease in Glx levels in nervous tissue has been observed in patients with Alzheimer’s disease (AD) and Parkinson’s disease, but also in healthy elderly individuals. It has been suggested that lower Glx levels may be associated with cognitive decline [31]. Moreover, a decrease in the Glx/tCr ratio was reported in the hypothalamus in patients with multiple sclerosis and was associated with worsening of cognitive processes [33].

Our study observed lower levels of NAA and TNAA in the pons, cerebellum, white matter, and thalamus, and reduced levels of NAAG in the cerebellum, white matter, and pons. NAA is a marker indicating neuronal integrity and has also been linked to mitochondrial function [34,35]. The decrease in the NAA/Cr (NAA/creatinine) ratio was described in the cerebellum in spinocerebellar ataxia type 27B (SCA27B), and in the pre-central area in Hereditary Spastic Paraplegias [22,36]. It has also been found that NAA levels slowly decline with age [32]. The reduction in TNAA, Glx, and Glu was also observed in patients with Parkinson’s disease [21]. Furthermore, patients with WFS have been observed to have decreased Ins levels in the cerebellum. Ins is a known marker for glial function and immunological processes [31,36]. Additionally, Kara et al. associated a decrement in the Ins/Cr ratio in the posterior cingulate gyrus and left hippocampus in AD patients with lower Mini-Mental State Examination scores, and the reduction in TNAA/mIns (myo-inositol) was linked to a decline in cognitive function [20].

Moreover, correlation analysis in our study revealed relationships between NAA levels and the left intracranial optic nerve, and between TNAA and optic tract thickness. Both of these parameters have already been reported as reduced in WFS, becoming recognised markers of neurodegeneration in these patients [28], further supporting the possibility of using NAA and TNAA to predict neurodegeneration in patients with WFS. It is also worth noting that optic nerve atrophy in WFS patients does not occur symmetrically at the same time; rather, the loss is gradual and affects different quadrants of the optic nerves at different times in individual patients [28,37]. The negative correlation between NAA and optic nerve thickness may be somewhat surprising. However, because optic nerve atrophy in Wolfram syndrome does not progress in a linear, continuous manner, this may, in our opinion, lead to an ambiguous effect on the direction of the correlation. Thus, NAA can be considered a marker of an ongoing neurodegenerative process rather than a process that has already ended. The analysis also revealed correlations between Ins and body weight in patients with WFS. Interestingly, a link between higher BMI and elevated Ins/Cr ratio has already been observed in obese individuals [38]. Moreover, our study found a correlation between GPC and BMI. Another study mentioned an increase in the GPC + PCh (phosphocholine)/Cr ratio in obese children with ADHD compared to children with ADHD of normal weight [39].

Next, the mitochondrial-to-nuclear DNA ratio was lower in our patients with WFS compared to healthy controls. Reduced mtDNA-CN levels have been reported in various neurodegenerative diseases, including AD, Parkinson’s disease, and Huntington’s disease, suggesting their role as prognostic biomarkers [24,40,41]. Decrease in mtDNA-CN was linked with a greater risk of AD development and transition from mild cognitive impairment to AD, making it a potential marker of disease progression [23]. Although a reduction in mtDNA copy number in WFS patients does not appear to be specific to this disease, it is worth noting that mitochondrial abnormalities in Wolfram syndrome may result from loss of function of the *WFS1* gene. This impairment disrupts interactions between the endoplasmic reticulum and mitochondria, a process shown to lead to mitochondrial dysfunction in cellular models of this syndrome [42]. On the other hand, reduced mtDNA-CN has also been reported in type 1 and type 2 diabetes, as well as in gestational diabetes [25,43,44]. In patients with long-standing type 1 diabetes, a reduction in mtDNA-CN was observed compared to controls, along with an association with treatment regimen and the presence of chronic diabetes complications [25]. Thus, given the decline in mtDNA-CN in both processes, the lower mtDNA-CN levels observed in WFS may reflect the cumulative effect of diabetes and accompanying neurodegeneration. Therefore, given this biological variability and the lack of significant correlations in our study between mtDNA-CN and MRI findings or clinical parameters, it is currently not possible, without a study that controls for the influence of diabetes itself, to identify mtDNA-CN as a marker of neurodegeneration in WFS.

The limitations of the study include the small study group size, due to the rarity of WFS, and the inability to perform correlation analysis in the control group. Unfortunately, we were unable to provide clinical data on healthy participants. Therefore, this study should be regarded as preliminary and exploratory. Further longitudinal studies involving larger patient cohorts, including patients with isolated type 1 diabetes, are needed to determine the usefulness of MRS and mtDNA-CN for predicting and monitoring neurodegeneration in patients with WFS.

## 5. Conclusions

In summary, the study provides a detailed MRS analysis showing reduced levels of selected metabolites across several brain regions and demonstrating decreased mtDNA-CN in WFS patients compared to healthy controls. However, our study is exploratory in nature and has allowed us to identify changes in neural tissue metabolites which—once validated and their translational significance established—may in the future serve as prognostic markers of neurodegeneration in patients with WFS. This would be of great significance for establishing and monitoring the course of clinical trials aimed at inhibiting neurodegeneration in these patients.

## Figures and Tables

**Table 1:** Correlations between MRI indicators of neurodegeneration and brain MRS metabolites and mtDNA-CN in patients with Wolfram syndrome. MRI—magnetic resonance imaging; MRS—magnetic resonance spectroscopy; mtDNA-CN—copy number of mitochondrial DNA; ON—optic nerve. Significant values of *p* and R are indicated in bold.

Parameter	Right Intraorbital ON, mm		Left Intraorbital ON, mm		Right Intracranial ON, mm		Left Intracranial ON, mm		Optic Chiasm, mm		Optic Tract, mm	
	*p*	R	*p*	R	*p*	R	*p*	R	*p*	R	*p*	R
Glu	0.587	−0.283	0.579	−0.289	0.430	−0.401	0.264	−0.544	0.064	0.786	0.242	0.566
Glx	0.521	−0.331	0.355	−0.463	0.506	−0.343	0.219	−0.588	0.180	0.630	0.214	0.593
GPC	0.389	−0.435	0.907	0.062	0.814	−0.125	0.874	0.084	0.146	0.669	0.682	−0.216
Ins	0.216	0.591	0.499	0.348	0.405	−0.421	0.223	−0.584	0.497	−0.350	0.446	0.389
NAA	0.781	0.147	0.761	−0.160	0.103	−0.724	**0.005**	**−0.939**	0.356	0.462	0.051	0.810
TNAA	0.675	0.220	0.805	−0.130	0.387	−0.436	0.066	−0.781	0.493	0.352	**0.011**	**0.913**
TCho	0.935	0.043	0.967	−0.022	0.965	−0.023	0.822	−0.117	0.222	0.585	0.248	0.560
Glc	0.089	0.746	0.577	0.289	0.185	−0.624	0.088	−0.747	0.957	0.029	0.055	0.802
NAAG	0.859	0.093	0.890	0.073	0.228	0.578	0.425	0.405	0.631	−0.250	0.947	0.034
lipa09	0.254	0.553	0.242	0.565	0.845	0.103	0.593	0.278	0.170	−0.640	0.514	−0.336
mtDNA-CN	0.569	0.296	0.126	0.695	0.617	−0.261	0.994	0.004	0.251	0.556	0.852	0.099

**Table 2:** Correlations between brain MRS metabolites and mtDNA-CN and clinical parameters in patients with Wolfram syndrome. MRS—magnetic resonance spectroscopy; mtDNA-CN—copy number of mitochondrial DNA; HbA1c—glycated haemoglobin; BMI—body mass index. Significant values of *p* and R are indicated in bold.

	Parameter	mtDNA-CN		Age at Disease Onset, Years		HbA1c, %		Glucose, mg/dL		Body Weight, kg		BMI	
Metabolite		*p*	R	*p*	R	*p*	R	*p*	R	*p*	R	*p*	R
Glu		0.721	0.139	0.118	−0.559	0.660	0.171	0.529	0.242	0.389	−0.327	0.311	−0.382
Glx		0.968	0.015	**0.042**	**−0.685**	0.330	0.368	0.311	0.381	0.508	−0.255	0.518	−0.250
GPC		0.423	0.306	0.647	0.178	0.185	−0.485	0.950	0.025	0.199	−0.472	**0.032**	**−0.711**
Ins		0.968	0.016	0.639	−0.182	0.136	0.537	0.712	0.144	**0.036**	**0.700**	0.053	0.659
NAA		0.997	0.001	0.077	−0.617	0.193	0.478	0.830	−0.084	0.868	−0.065	0.927	−0.036
TNAA		0.815	0.092	0.359	−0.348	0.675	0.163	0.373	−0.339	0.804	−0.097	0.893	−0.053
TCho		0.984	−0.007	0.472	0.276	0.225	−0.449	0.979	0.009	0.731	−0.134	0.703	−0.148
Glc		0.818	0.090	0.719	−0.140	0.104	0.577	0.433	−0.300	0.228	0.447	0.086	0.603
NAAG		0.652	0.174	0.301	0.388	0.163	−0.506	0.158	−0.512	0.846	−0.075	0.917	−0.040
lipa09		0.488	−0.266	**0.015**	**0.768**	0.566	−0.221	0.886	0.056	0.053	0.658	0.104	0.575

## Data Availability

The data presented in this study are available on request from the corresponding author due to patients’ privacy.

## Supplementary Materials

The following supporting information can be downloaded at: https://www.mdpi.com/article/10.3390/metabo16040281/s1, Supplementary Table S1. Sequences of primers and probes used for quantitative PCR analysis of mitochondrial and nuclear DNA., Supplementary Table S2. Mean metabolite values and *p* values adjusted by the false discovery rate (FDR) correction for each brain region. and Supplementary Figure S1. Noise-free fits to the spectral peaks from all regions of interest: cerebellum, hippocampus, pons, thalamus and white matter. Supplementary Figure S2. Spectral fitting results of cerebellum. The baseline and metabolite fits are shown in green, the model signal in red, and the residual water signal (uppermost signal in the plot) together with the processed signal in black. Supplementary Figure S3. Spectral fitting results of cerebellum. The baseline and metabolite fits are shown in green, the model signal in red, and the residual water signal (uppermost signal in the plot) together with the processed signal in black. The baseline is suppressed. Supplementary Figure S4. Spectral fitting results of hippocampus. The baseline and metabolite fits are shown in green, the model signal in red, and the residual water signal (uppermost signal in the plot) together with the processed signal in black. Supplementary Figure S5. Spectral fitting results of hippocampus. The baseline and metabolite fits are shown in green, the model signal in red, and the residual water signal (uppermost signal in the plot) together with the processed signal in black. The baseline is suppressed. Supplementary Figure S6. Spectral fitting results of pons. The baseline and metabolite fits are shown in green, the model signal in red, and the residual water signal (uppermost signal in the plot) together with the processed signal in black. Supplementary Figure S7. Spectral fitting results of pons. The baseline and metabolite fits are shown in green, the model signal in red, and the residual water signal (uppermost signal in the plot) together with the processed signal in black. The baseline is suppressed. Supplementary Figure S8. Spectral fitting results of thalamus. The baseline and metabolite fits are shown in green, the model signal in red, and the residual water signal (uppermost signal in the plot) together with the processed signal in black. Supplementary Figure S9. Spectral fitting results of thalamus. The baseline and metabolite fits are shown in green, the model signal in red, and the residual water signal (uppermost signal in the plot) together with the processed signal in black. The baseline is suppressed. Supplementary Figure S10. Spectral fitting results of white matter. The baseline and metabolite fits are shown in green, the model signal in red, and the residual water signal (uppermost signal in the plot) together with the processed signal in black. Supplementary Figure S11. Spectral fitting results of white matter. The baseline and metabolite fits are shown in green, the model signal in red, and the residual water signal (uppermost signal in the plot) together with the processed signal in black. The baseline is suppressed.

## Abbreviations

The following abbreviations are used in this manuscript:

AD	Alzheimer’s disease
BMI	body mass index
Cr	creatinine
GCF	gingival crevicular fluid
Glc	Glucose
GPC	glycerophosphocholines
Glu	Glutamate
Glx	Glutamate and Glutamine
HbA1c	glycated hemoglobin
Ins	myo-inositol
KNN	k-Nearest Neighbors
LC-MS	Liquid chromatography–Mass spectrometry
Lipa09	mobile lipids
MRI	magnetic resonance images
MRS	magnetic resonance spectroscopy
mtDNA-CN	copy number of mitochondrial DNA
NAA	N-acetylaspartate
NAAG	N-acetylaspartylglutamate
OCT	optical coherence tomography
PCh	phosphocholine
SCA27B	spinocerebellar ataxia type 27B
Tcho	total Choline
TCr	total creatine
TNAA	total N-acetylaspartate
WFS	Wolfram syndrome
