# Supplementary material for: Selected Brain Metabolites and Mitochondrial DNA Copy Number as Potential Markers of Ongoing Neurodegeneration in Patients with Wolfram Syndrome
Source: Metabolites. 2026 Apr 20;16(4):281. doi: 10.3390/metabo16040281 (PMC13117842; doi:10.3390/metabo16040281)
Supplement: Supplementary file 1 [file metabolites-16-00281-s001.zip › metabolites-4227927-supplementary figures.pdf]

Supplementary figure S1. Noise-free fits to the spectral peaks from all regions of interest: cerebellum, hippocampus, pons, thalamus and white matter.

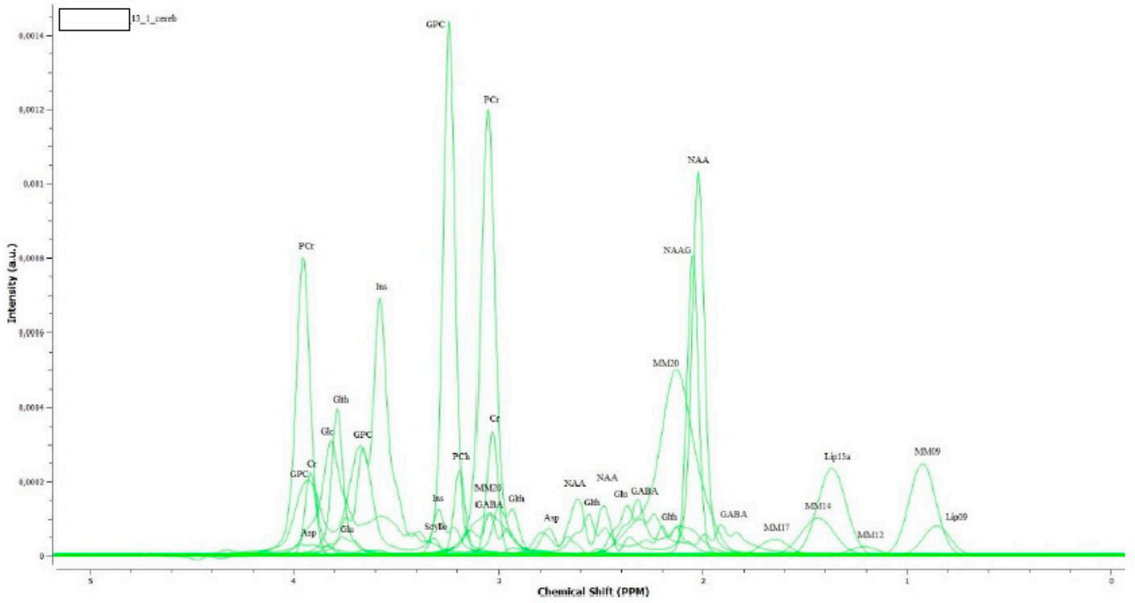

Supplementary figure S2. Spectral fitting results of cerebellum. The baseline and metabolite fits are shown in green, the model signal in red, and the residual water signal (uppermost signal in the plot) together with the processed signal in black.

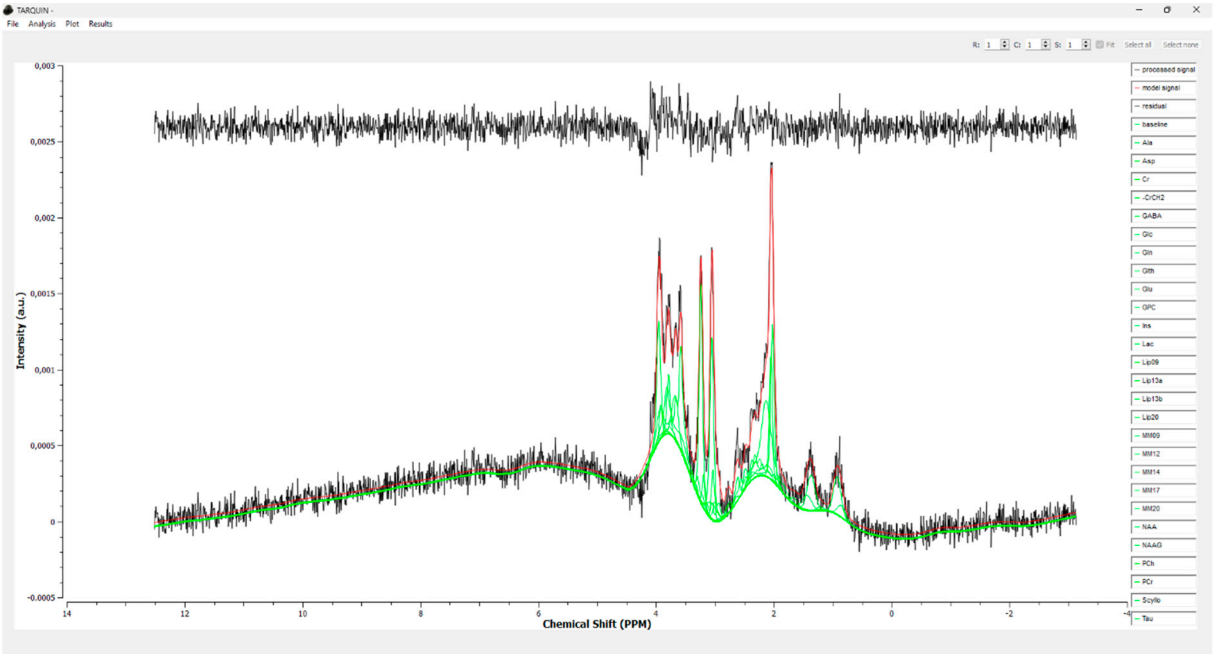

Supplementary figure S3. Spectral fitting results of cerebellum. The baseline and metabolite fits are shown in green, the model signal in red, and the residual water signal (uppermost signal in the plot) together with the processed signal in black. The baseline is suppressed.

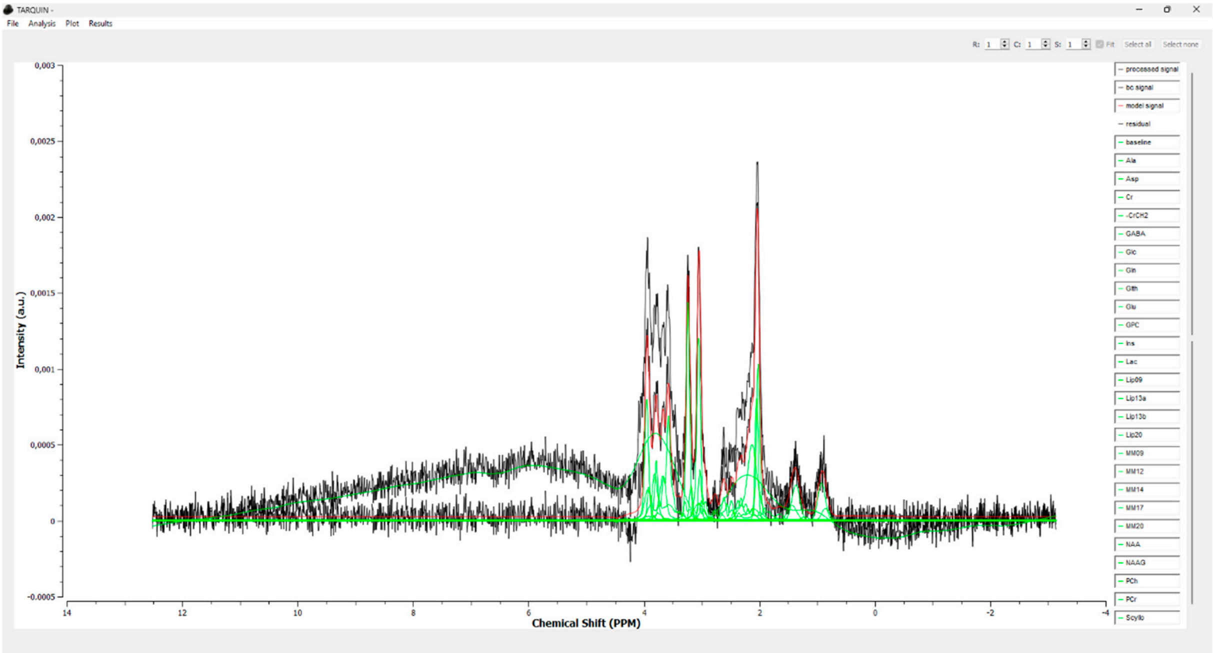

Supplementary figure S4. Spectral fitting results of hippocampus. The baseline and metabolite fits are shown in green, the model signal in red, and the residual water signal (uppermost signal in the plot) together with the processed signal in black.

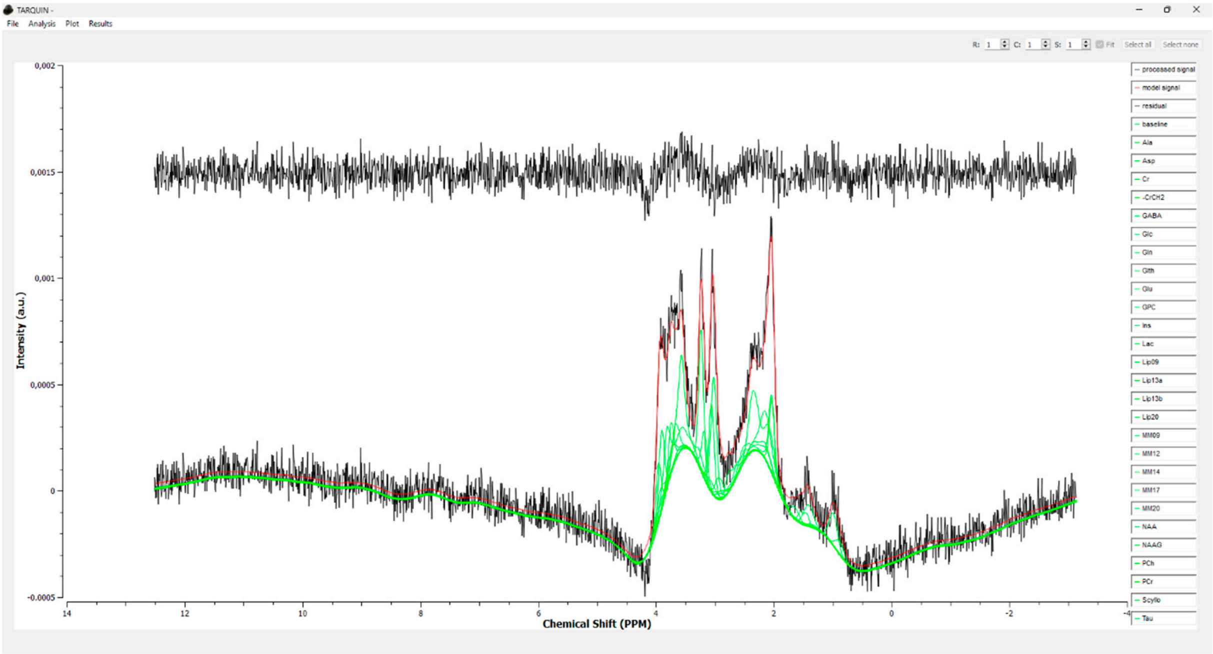

Supplementary figure S5. Spectral fitting results of hippocampus. The baseline and metabolite fits are shown in green, the model signal in red, and the residual water signal (uppermost signal in the plot) together with the processed signal in black. The baseline is suppressed.

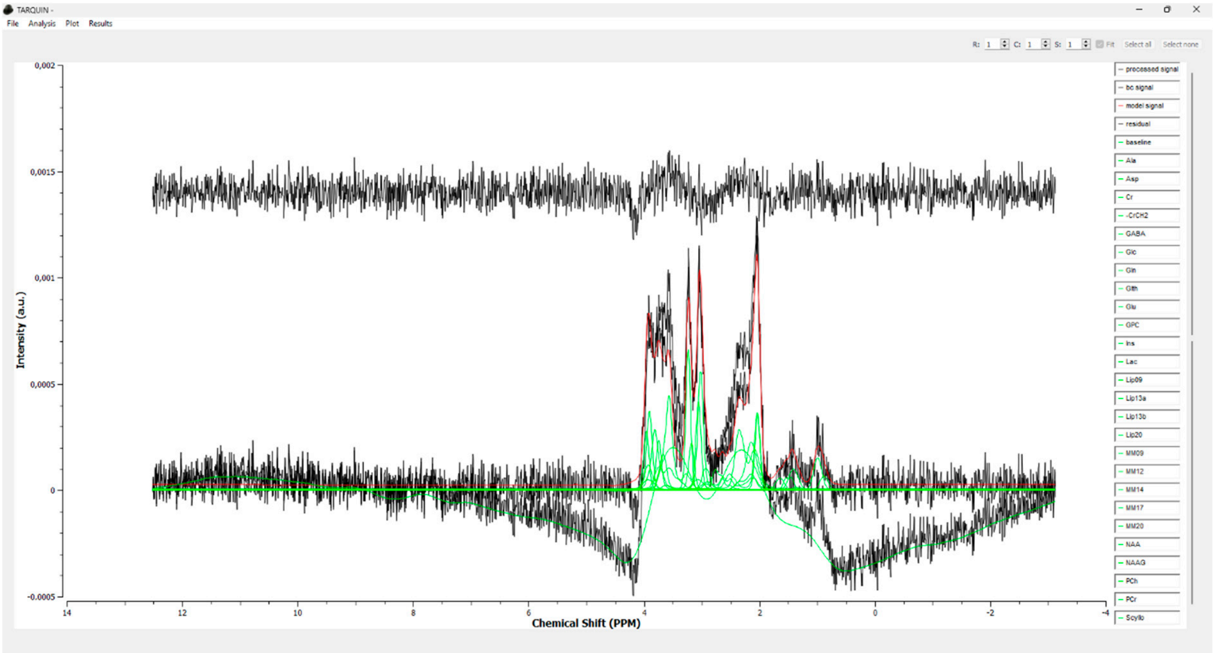

Supplementary figure S6. Spectral fitting results of pons. The baseline and metabolite fits are shown in green, the model signal in red, and the residual water signal (uppermost signal in the plot) together with the processed signal in black.

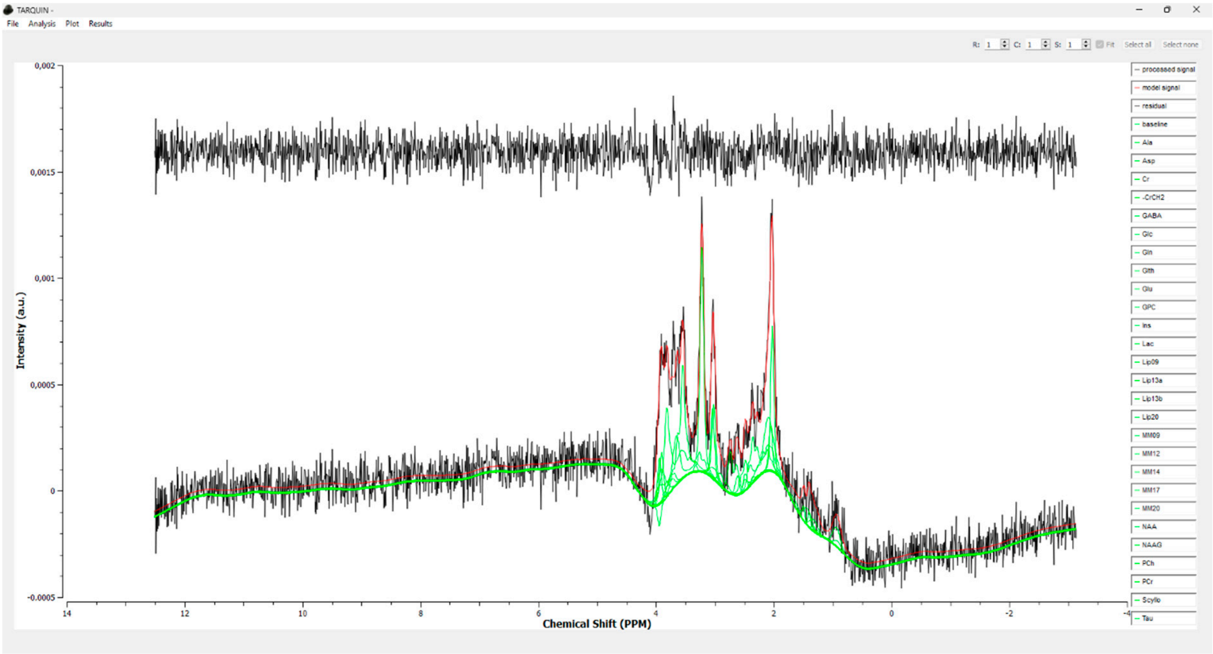

Supplementary figure S7. Spectral fitting results of pons. The baseline and metabolite fits are shown in green, the model signal in red, and the residual water signal (uppermost signal in the plot) together with the processed signal in black. The baseline is suppressed.

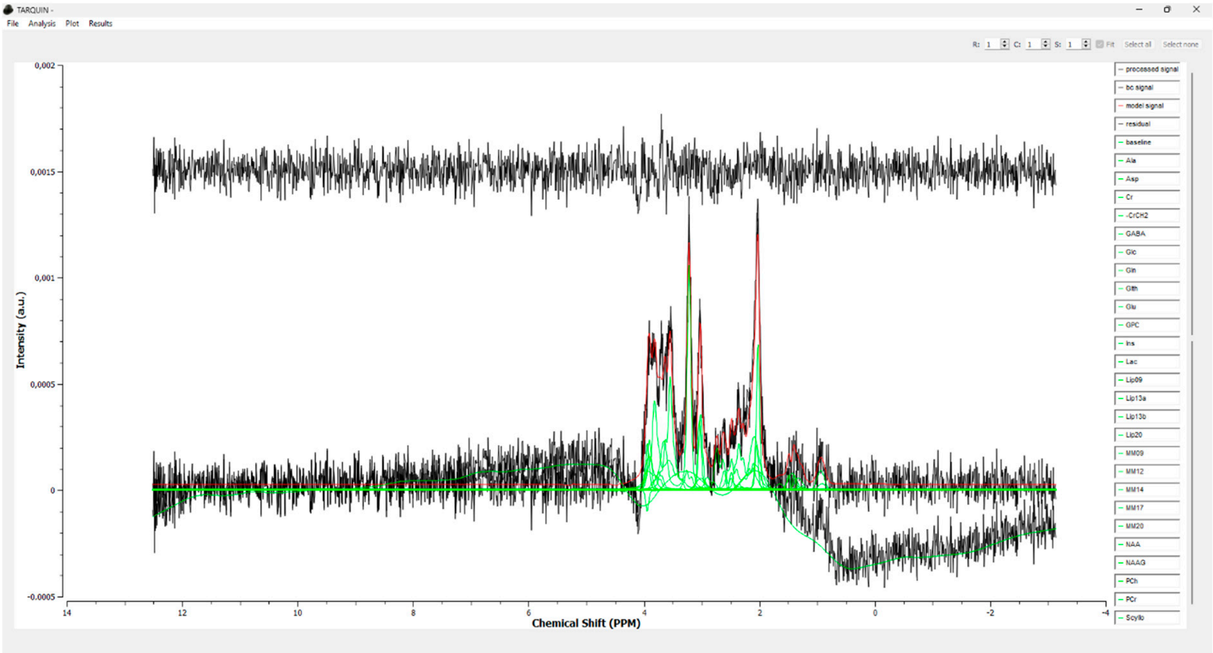

Supplementary figure S8. Spectral fitting results of thalamus. The baseline and metabolite fits are shown in green, the model signal in red, and the residual water signal (uppermost signal in the plot) together with the processed signal in black.

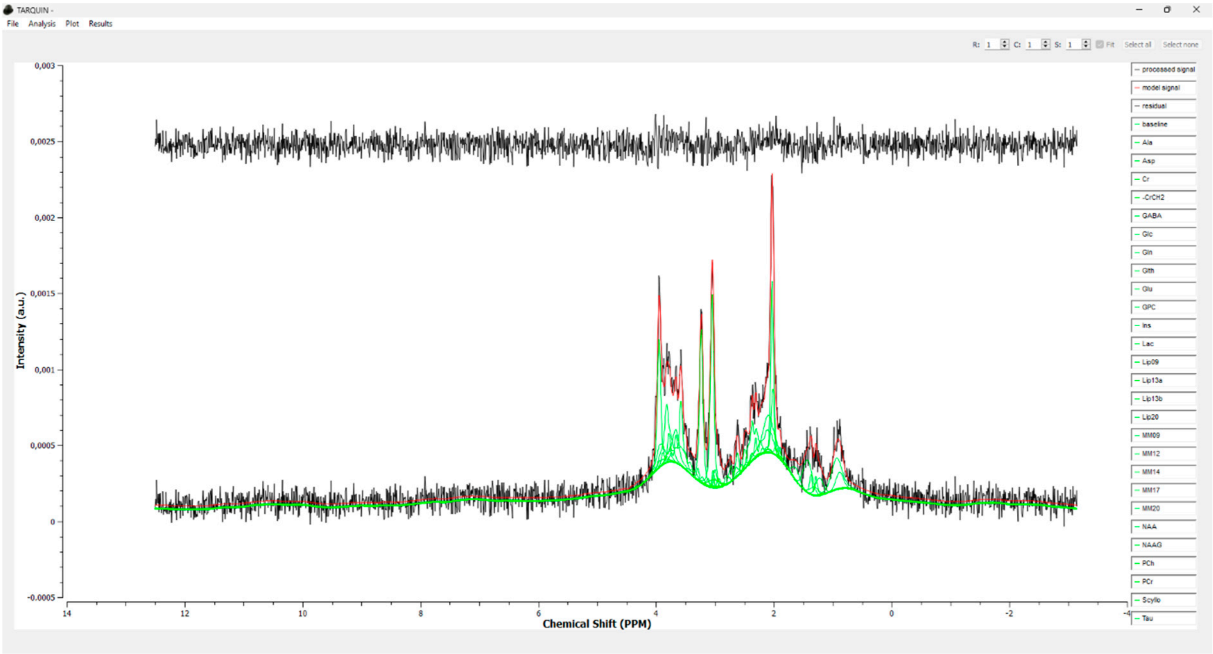

Supplementary figure S9. Spectral fitting results of thalamus. The baseline and metabolite fits are shown in green, the model signal in red, and the residual water signal (uppermost signal in the plot) together with the processed signal in black. The baseline is suppressed.

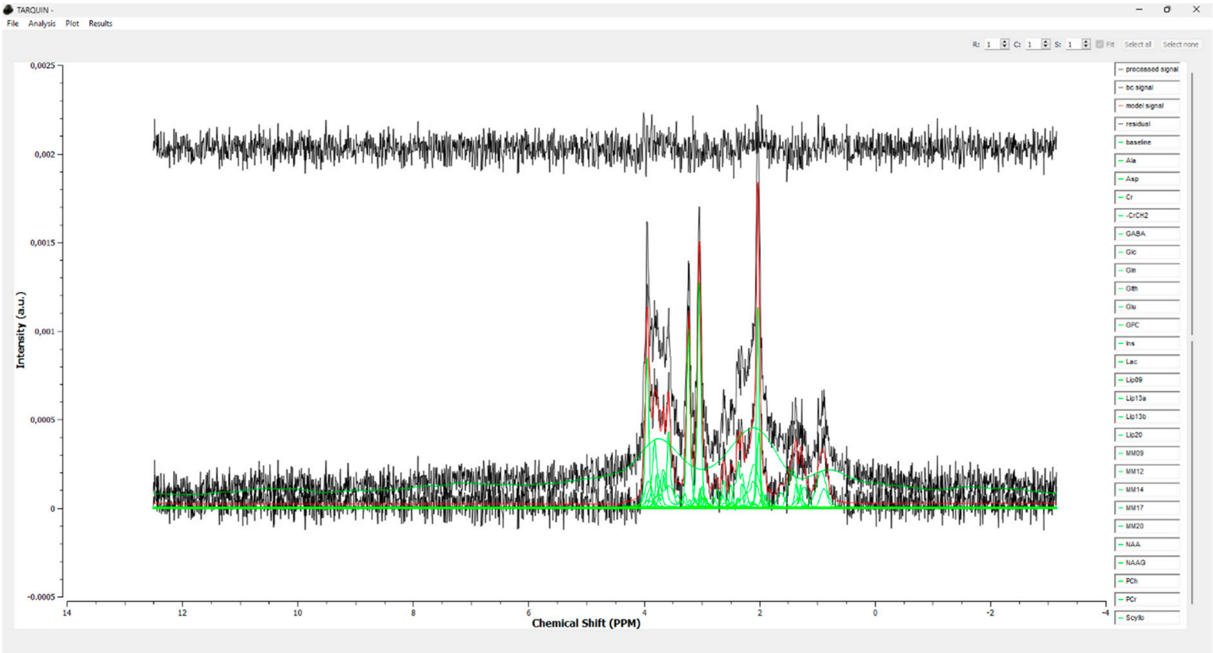

Supplementary figure S10. Spectral fitting results of white matter. The baseline and metabolite fits are shown in green, the model signal in red, and the residual water signal (uppermost signal in the plot) together with the processed signal in black.

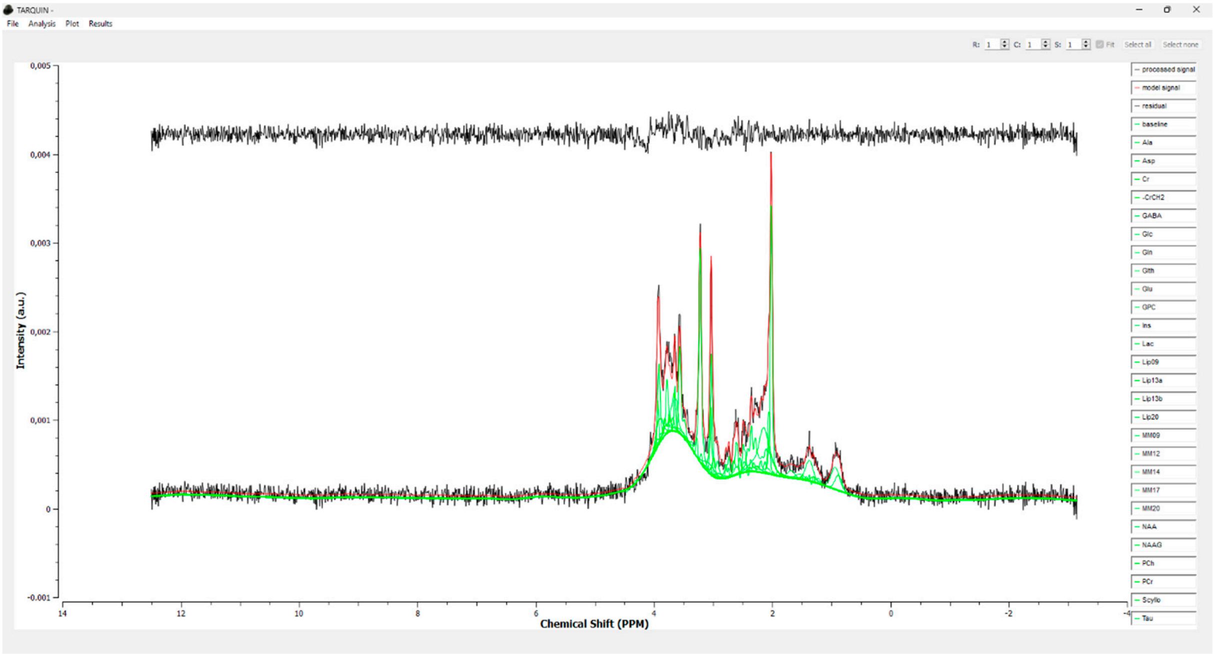

Supplementary figure S11. Spectral fitting results of white matter. The baseline and metabolite fits are shown in green, the model signal in red, and the residual water signal (uppermost signal in the plot) together with the processed signal in black. The baseline is suppressed.

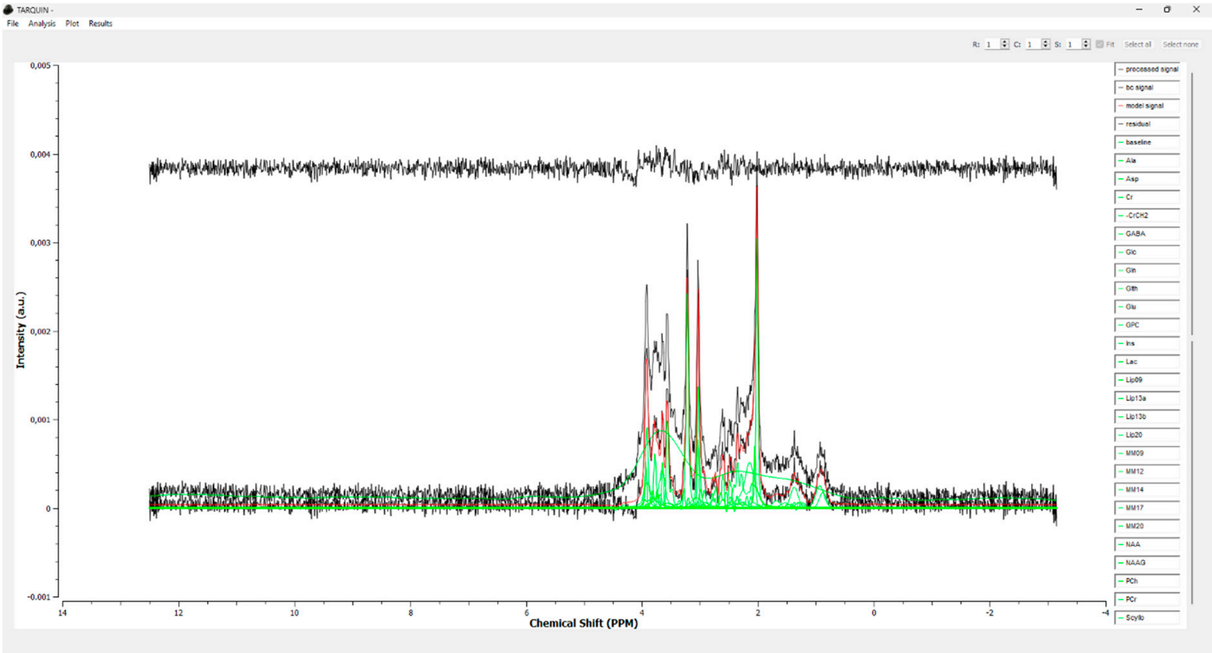
TARQUIN -
File
Analysis
Plot
Results
R: 1
C: 1
S: 1
Fit
Select all
Select none
Intensity (a.u.)
0,005
0,004
0,003
0,002
0,001
0
-0.001
14
12
10
8
6
4
2
0
-2
-4
Chemical Shift (PPM)
processed signal
bc signal
model signal
residual
baseline
Ala
Asp
Cr
-CrCH2
GABA
Glc
Gln
Glth
Glu
GPC
Ins
Lac
Lip09
Lip13a
Lip13b
Lip20
MM09
MM12
MM14
MM17
MM20
NAA
NAAG
PCh
PCr
Scyllo
