# Supplementary material for: Selected Brain Metabolites and Mitochondrial DNA Copy Number as Potential Markers of Ongoing Neurodegeneration in Patients with Wolfram Syndrome
Source: Metabolites. 2026 Apr 20;16(4):281. doi: 10.3390/metabo16040281 (PMC13117842; doi:10.3390/metabo16040281)
Supplement: Supplementary file 1 [file metabolites-16-00281-s001.zip › Supplementary Table S1_.pdf]

Supplementary Table S1. Sequences of primers and probes used for quantitative PCR analysis of mitochondrial and nuclear DNA.

Forward primer_mtDNA	ATTATACCCACACCCACCCAAGAAC
Reverse primer_mtDNA	ATGGGTACAATGAGGAGTAGGAGGT
Probe_mtDNA	CCGGGCTCTGCCAT
Forward primer_nuclearDNA	TGCTGTCTCCATGTTTGATGTATCT
Reverse primer_nuclearDNA	TCTCTGCTCCCCACCTCTAAGT
Probe_nuclearDNA	CTTAGAGGTGGGGAGCAGAG
